# Improving HIV Surveillance Data for Public Health Action in Washington, DC: A Novel Multiorganizational Data-Sharing Method

**DOI:** 10.2196/publichealth.5317

**Published:** 2016-01-15

**Authors:** Joanne Michelle F Ocampo, JC Smart, Adam Allston, Reshma Bhattacharjee, Sahithi Boggavarapu, Sharon Carter, Amanda D Castel, Jeff Collmann, Colin Flynn, Auntré Hamp, Diana Jordan, Seble Kassaye, Michael Kharfen, Garret Lum, Raghu Pemmaraju, Anne Rhodes, Jeff Stover, Mary A Young

**Affiliations:** ^1^ The Office of the Senior Vice President for Research Georgetown University Washington, DC United States; ^2^ Department of Computer Science Georgetown University Washington, DC United States; ^3^ The HIV/AIDS, Hepatitis, STD and TB Administration District of Columbia Department of Health Washington, DC United States; ^4^ Prevention and Health Promotion Administration Maryland Department of Health and Mental Hygiene Baltimore, MD United States; ^5^ Division of Disease Prevention Virginia Department of Health Richmond, VA United States; ^6^ Milken Institute School of Public Health Department of Epidemiology and Biostatistics The George Washington University Washington, DC United States; ^7^ The HIV/AIDS, Hepatitis, STD and TB Administration Washington District of Columbia Department of Health Washington, DC United States; ^8^ The Washington DC Metropolitan Women’s Interagency HIV Study Georgetown University Medical Center Washington, DC United States; ^9^ University Information Services Georgetown University Washington, DC United States

**Keywords:** HIV, surveillance, data sharing, public health, technology

## Abstract

**Background:**

The National HIV/AIDS Strategy calls for active surveillance programs for human immunodeficiency virus (HIV) to more accurately measure access to and retention in care across the HIV care continuum for persons living with HIV within their jurisdictions and to identify persons who may need public health services. However, traditional public health surveillance methods face substantial technological and privacy-related barriers to data sharing.

**Objective:**

This study developed a novel data-sharing approach to improve the timeliness and quality of HIV surveillance data in three jurisdictions where persons may often travel across the borders of the District of Columbia, Maryland, and Virginia.

**Methods:**

A deterministic algorithm of approximately 1000 lines was developed, including a person-matching system with Enhanced HIV/AIDS Reporting System (eHARS) variables. Person matching was defined in categories (from strongest to weakest): exact, very high, high, medium high, medium, medium low, low, and very low. The algorithm was verified using conventional component testing methods, manual code inspection, and comprehensive output file examination. Results were validated by jurisdictions using internal review processes.

**Results:**

Of 161,343 uploaded eHARS records from District of Columbia (N=49,326), Maryland (N=66,200), and Virginia (N=45,817), a total of 21,472 persons were matched across jurisdictions over various strengths in a matching process totaling 21 minutes and 58 seconds in the privacy device, leaving 139,871 uniquely identified with only one jurisdiction. No records matched as medium low or low. Over 80% of the matches were identified as either exact or very high matches. Three separate validation methods were conducted for this study, and they all found ≥90% accuracy between records matched by this novel method and traditional matching methods.

**Conclusions:**

This study illustrated a novel data-sharing approach that may facilitate timelier and better quality HIV surveillance data for public health action by reducing the effort needed for traditional person-matching reviews without compromising matching accuracy. Future analyses will examine the generalizability of these findings to other applications.

## Introduction

The US National HIV/AIDS Strategy has an increased focus on retention and re-engagement in medical care and therefore includes an action step to “strengthen the timely availability and use of data” [1]. It calls upon human immunodeficiency virus (HIV) surveillance programs to better measure the continuum of care for persons living with HIV (PLWH) in their jurisdictions and also to identify individuals who are in need of public health services aimed at improving linkage, retention, and viral suppression for PLWH. These activities are often described as data to care (D2C), as they utilize HIV surveillance data for public health action [2]. To effectively perform these D2C activities, HIV surveillance programs need more complete, accurate, and timely measures of PLWH currently in their jurisdictions, and information on whether and where they are receiving care, and their health status (eg, antiretroviral therapy use, CD4, and viral load measurements). However, several challenges that affect HIV surveillance programs’ D2C activities still remain, including technical (eg, data format, missing data caused by case migration across jurisdictions, out-of-jurisdiction care utilization by HIV cases, and incomplete reporting by out-of-jurisdiction laboratories used by in-jurisdiction HIV care providers), motivational (opportunity cost), economic (ownership/copyright), privacy, and ethical barriers [3,4].

Cross-jurisdictional notification of HIV cases, as directed by the Centers for Disease Control and Prevention (CDC) and the Council of State and Territorial Epidemiologists, was designed to ensure that all HIV cases are reported, but it does not function well at tracking PLWH and their residence data over time or reporting all instances of HIV care. The existing national de-duplication process, the Routine Interstate Duplicate Review (RIDR), is time- and resource-intensive, operates with a significant delay between case report and duplicate resolution, and most importantly, focuses mainly on residence at initial diagnosis [5]. RIDR therefore does not adequately serve as an effective tool to notify jurisdictions of updates to vital status or address information, or to provide updated information on migration or participation in out-of-jurisdiction care.

Effective utilization of big data can now facilitate timelier HIV surveillance. Previous studies have demonstrated the potential of effective data sharing technologies. In 2008, Pacheco et al developed a hierarchical deterministic linkage technique and fully automated matching algorithm for recovering the vital status of people from different cohort data in Brazil [6]. They did so to facilitate investigators’ efforts at finding people lost to follow-up in mortality databases. Effective technological advances can help public health officials develop more up-to-date models of HIV diagnosis, linkage to, and retention in care in the United States and elsewhere.

The US mid-Atlantic region experiences some of the highest HIV prevalence rates among key population groups in the country and is therefore critical to the national response to HIV [7]. Health officials in this region have long hypothesized movement of PLWH across the jurisdictions of the District of Columbia (DC), the State of Maryland (MD), and the Commonwealth of Virginia (VA), but traditional barriers to data sharing have prevented comprehensive examinations of this phenomena. In addition, there has been a longstanding dialogue about HIV in the Washington, DC, metropolitan region between Georgetown University and the DC Department of Health on elements of clinical care, longitudinal research, student internships, project planning, and many shared speaking engagements, and also collaboration through the District of Columbia Center for AIDS Research (DC-CFAR). Therefore, in January 2013, health officials from DC, MD, and VA and others met at Georgetown University to discuss regional sharing of HIV/AIDS data in the Washington DC metropolitan region (including areas in DC, MD, and VA). During the meeting, they identified the pressing need for a novel and more timely approach to sharing HIV surveillance data for D2C activities in this region. They emphasized that any such approach must account for the highly private and sensitive nature of public health data. Following these aims, this study used a novel data-sharing approach to examine cross-jurisdictional person-matches of PLWH among the public health departments of DC, MD, and VA.

## Methods

### Study Population

The study population included all persons with valid Enhanced HIV/AIDS Reporting System (eHARS) records in DC, MD, and VA databases from 1981 to 2015. eHARS is a Web-based data system with an SQL-server back-end that is provided by the CDC to all jurisdictions that collect HIV surveillance data. Most jurisdictions maintain their HIV surveillance data in eHARS, and all jurisdictions submit monthly data to the CDC through eHARS. SAS versions 9.3 and 9.4 were used to preprocess data from eHARS into a standardized format across jurisdictions. These criteria excluded noncases, perinatal HIV exposure records, records still under investigation, and not yet designated as cases, and cases with errors, required fields missing, or marked for deletion/purging.

### Institutional Review Board and Privacy

The study was reviewed by the Georgetown University Institutional Review Board and was deemed exempt because of the experimental design and computer technology that specifically prevents persons from seeing person-identifiable information, including the Health Insurance Portability and Accountability Act (HIPAA)-protected information (see Multimedia Appendix 1). For pre- and post-experiment disaggregated data processing and analysis of HIPAA-protected information, each public health department followed internal procedures specific to their jurisdiction. A collaboratively developed project-specific data security and confidentiality procedures manual was applied in this study and was signed by all jurisdictions and Georgetown University representatives.

### Algorithm, Hardware, and System Configuration

Ada is a structured programming language defined by ISO/IEC 8652:2012. Originally funded by the United States Department of Defense in 1973 to supersede hundreds of programming languages then in use, Ada was specifically designed for high integrity applications where code safety and reliability is paramount. The Ada programming language was selected for algorithm implementation here because of its unambiguous semantics, extremely strong type and constraint checking, exception protections, and overall reliability philosophy. A deterministic treatment sustainment algorithm of approximately 1000 lines and a technologically robust computer (ie, the privacy device) and physically secure environment were used in this study (see Multimedia Appendix 1; [8]) following a privacy technology approach previously described [9]. For identifying false positives (ie, people who matched across jurisdictions but should not have been matched) or locating false negatives (ie, people who were not matched across jurisdictions but should have been matched), manual case investigations of suspected cases were conducted by each jurisdiction. No direct access existed between jurisdictions’ eHARS servers and the privacy device. Instead, jurisdictions posted information onto a secure file transfer protocol site that then sent information (synthetic test and real eHARS data) to the privacy device.

### Algorithm Testing and Verification Using Synthetic Data

Verification of the privacy device system was undertaken using conventional component testing methods, manual code inspection, and comprehensive output file examination. A separate, but similar computer from the production company was used for testing and verification of the program described above. Facilitated by the participating jurisdictions and the CDC, a large corpus of synthetic test data were made available to test the algorithm. The majority of programming errors were identified in the conversion process of external data files. Once ingested and represented within Ada's strongly typed framework, no errors that would result in program failure were identified. Thorough testing of the algorithm uncovered an incorrect assumption about the initial value of the variable at the beginning of a programming loop under wildcard matching conditions. This error was detected and corrected with the aid of Ada 2012 preconditions and inline assertions. The matching algorithm categories included (from strongest to weakest): exact, very high, high, medium high, medium, medium low, low, and very low (see Table 1).

**Table 1:** Overview of categories and definitions used in the study’s person-matching algorithm.

Matching categories	Variable definitions^a^
Exact	if m.last_name and m.first_name and m.dob and m.ssn and m.sex and m.race then m.score := exact;
Very high	elsif (m.last_name and m.first_name and m.dob and m.sex) or m.ssn then m.score := very_high;
High	elsif m.last_name and m.first_name and m.dob and (m.sex or m.race) then m.score := high;
Medium high	elsif m.last_name and m.first_soundex and m.dob and m.sex then m.score := medium_high;
Medium (1st definition)	elsif m.last_name and m.dob and m.sex and m.race then m.score:= medium;
Medium (2nd definition)	elsif m.last_soundex and m.first_soundex and m.dob and (m.sex or m.race) then m.score := medium;
Medium low	elsif m.last_soundex and m.first_soundex and m.partial_dob and m.partial_ssn and (m.sex or m.race) then m.score := medium_low;
Low	elsif m.last_soundex and (m.partial_dob and m.partial_ssn) and (m.sex or m.race) then m.score := low;
Very low	elsif m.last_soundex and (m.partial_dob or m.partial_ssn) then m.score := very_low;

^a^Last name=Last name of PLWH in eHARS person file; First name=First name of PLWH in eHARS person file; DOB=Date of birth of PLWH in eHARS person file; SSN=Social Security Number of PLWH in eHARS person; Race=hierarchical race/ethnicity assignment for PLWH in eHARS person-view; Soundex=Soundex is a phonetic, alphanumeric code created by converting a name into an index letter and a 3-digit code. The index letter is the first letter of the name. The 3-digit code is calculated from the remaining letters of the name, based on rules found in the eHARS Technical Guidance. There is a Soundex variable for first name and a Soundex for last Name.

### Output Validation

Three separate validation methods were used to verify the findings of the privacy device to ensure validity across all jurisdictions.

DC utilized Link Plus software to validate the matching algorithm returns using the following parameters selected on the basis of being frequently used for other internal matching purposes: first name, last name, date of birth, and social security number. The Link Plus–selected matching method for first name and last name was “exact” and the matching method for date of birth and social security number was “generic string.” Generic string was selected for date of birth and social security number to allow for matches of partial dates of birth and social security number. A minimum selection score of zero was selected in order to maximize the number of potential matches that would be manually reviewed. Link Plus selected potential matches at 95% confidence interval based on the parameters specified, and cases that were not within the confidence interval for the four selected parameters were deemed nonmatches by the program and were exported and not reviewed. For cases that were reviewed, a hierarchy was used to determine where a case was a match: (1) all cases with exact matches with all four parameters were deemed matches; (2) all cases with matching social security numbers (even in cases where there were discrepancies in the other three parameters) were deemed matches; (3) for all four parameters, a fuzzy match approach was taken to determine if cases were matches, but typographical errors were made in data entry or in the data received by the health department; and (4) cases that were selected as nonmatches during manual review, but that had the previously described RIDR table or ID table (created when eHARS is exported out as a dataset, where unique identifiers are stored) information with matching STATENO (state number) were considered a match.

For accepted matches, MD used matches where the STATENO from other jurisdictions matched the previously collected STATENO from that jurisdiction already in the MD eHARS database, both from RIDR and ID tables. In cases where there was a conflict between the RIDR and the ID tables, manual review of the matching variables (name, DOB, SSN, race, sex) was conducted before the match was accepted or rejected. Any matches that had nonmatching STATENOs were checked to see if there was an apparent error in the MD version of the STATENOs. If there was, manual review of the matching variables was done to determine an acceptable match using the STATENO provided by the other jurisdiction as the correct STATENO (meaning the STATENO that MD had in their eHARS database was an error). For matches with missing STATENO in the MD database, manual review of the matching variables was done to establish if they were acceptable matches or not. Manual review was done for all exact and very high matching categories and on a 5% sample (no less than 10) from each of high, medium high, medium, and very low categories.

For the last validation method, VA split the DC/MD-VA matched dataset into VA, MD, and DC datasets by STATENO and matched with their respective states’ patient identifying data. The match was based on VA and the respective state’s STATENO. The datasets were then run through the Link King software that identified potential matches between the DC/MD and VA data. Potential matches were identified based on first name, last name, date of birth, race, and social security number. Potential matches were assigned a certainty level from 1 to 4 (strongest to weakest). Observations with no potential matches identified were left unmatched for manual review. The Link King results were matched again by STATENOs to the privacy device match level. A simple random sample from each privacy device match level was taken. A 25% sample was taken from the exact and very high match levels, a 30% sample from the high match level, a 40% sample from the medium high match level, and a 50% sample from the medium and very low match levels. The Link King software did identify a few DC/MD cases that were duplicates within the same DC/MD datasets. While noted, only one of the cases was included in the analysis dataset. There were several cases in the DC and MD datasets that were matched to more than one STATENO; however, only the matches that were accounted for by Link King and were the highest match level were incorporated in the analyses. The review indicated that over 90% of matches in the exact, very high, and high categories were affirmed by Link King to be strong matches.

## Results

This study found that from 1981 to 2015, a total of 21,472 persons were matched in eHARS databases across DC, MD, and VA over various strengths in a matching process totaling 21 minutes and 58 seconds in the privacy device, leaving 139,871 uniquely identified with only one jurisdiction (see Table 2). More than 80% were high-level matches, including 5933 exact matches, and 11,590 very high matches. Overall, more than 90% of matched records across all three jurisdictions were considered accurate matches after the three separate validation methods were applied in each jurisdiction (see Tables 3-5).

**Table 2:** Overview of person matches in eHARS databases across DC, MD, and VA from 1981 to 2015.

Person matches across jurisdictions	Exact	Very high	High	Medium high	Medium	Very low	Total
DC-MD^a^	4013	5907	53	268	645	482	11,368
MD-VA^b^	856	2343	11	117	377	865	4569
VA-DC^c^	1064	3340	15	149	438	529	5535
Total	5933	11,590	79	534	1460	1876	21,472

^a^DC-reported MD matches were equal to MD-reported DC matches.

^b^MD-reported VA matches were equal to VA-reported MD matches.

^c^VA-reported DC matches were equal to DC-reported VA matches.

**Table 3:** DC validation results.

		Nonmatch		Match		Total	
		N	%	N	%	N	%
**District of Columbia/Maryland**							
	Exact	0	0.0	4009	100.0	4009	100.0
	Very High	264	4.5	5560	95.5	5824	100.0
	High	0	0.0	52	100.0	52	100.0
	Medium High	3	1.1	264	98.9	267	100.0
	Medium	178	28.0	457	72.0	635	100.0
	Very Low	329	69.9	142	30.2	471	100.0
	Total	774	6.9	10484	93.1	11,258	100.0
**District of Columbia/Virginia**							
	Exact	0	0.0	1067	100.0	1067	100.0
	Very High	33	1.0	3286	99.0	3319	100.0
	High	0	0.0	13	100.0	13	100.0
	Medium High	5	3.4	144	96.6	149	100.0
	Medium	91	20.9	344	79.1	435	100.0
	Very Low	401	79.1	106	20.9	507	100.0
	Total	530	9.7	4960	90.4	5490	100.0

**Table 4:** MD validation results^d^.

		Nonmatch		Match		Total	
		N	%	N	%	N	%
**Maryland/District of Columbia**							
	Exact	0	0.0	4030	100.0	4030	100.0
	Very High	24	0.4	5846	99.2	5870	100.0
	High	0	0.0	52	100.0	52	100.0
	Medium High	0	0.0	272	100.0	272	100.0
	Medium	431	67.5	N/A	73.9	638	100.0
	Very Low	441	94.4	N/A	28.6	467	100.0
	Total				98.6	11,329	100.0
**Maryland/Virginia**							
	Exact	0	0.0	855	100.0	855	100.0
	Very High	10	0.4	2336	99.7	2344	100.0
	High	0	0.0	11	100.0	11	100.0
	Medium High	0	0.0	118	100.0	118	100.0
	Medium	292	77.5	N/A	90.3	377	100.0
	Very Low	827	96.3	N/A	15.3	858	100.0
	Total				97.7	4563	100.0

^d^Since a 5% random sample was used to manually review Medium & Very Low categories, exact numbers (N) of matches could not be shown in this table.

**Table 5:** VA validation results.

		Nonmatch		Match		Total	
		N	%	N	%	N	%
**Virginia/Maryland**							
	Exact	0	0.0	214	100.0	214	100.0
	Very High	21	3.6	562	96.4	583	100.0
	High	0	0.0	4	100.0	4	100.0
	Medium High	3	6.4	44	93.6	47	100.0
	Medium	98	53.3	86	46.7	184	100.0
	Very Low	400	98.0	8	2.0	408	100.0
	Total	522	36.3	918	63.8	1440	100.0
**Virginia/District of Columbia**							
	Exact	0	0.0	264	100.0	264	100.0
	Very High	14	1.7	802	98.3	816	100.0
	High	0	0.0	4	100.0	4	100.0
	Medium High	4	6.9	54	93.1	58	100.0
	Medium	69	33.3	138	66.7	207	100.0
	Very Low	199	87.7	28	12.3	227	100.0
	Total	286	18.1	1290	81.9	1576	100.0

## Discussion

### Application to HIV Surveillance Data and Public Health Action

Public health resources are limited and enhancements in surveillance data can assist with improved utilization of such resources. More specifically, improvements in the accuracy and timeliness of surveillance data is critical for D2C activities because it reduces the time and effort expended by staff in tracking down persons moving across jurisdictions who do not require assistance re-engaging in care. Using the novel data-sharing approach developed in this study, DC, MD, and VA were able to identify the number of person matches of people with known HIV status from 1981 to 2015 across their eHARS databases in a relatively short amount of time. This allowed more detailed follow-up data exchanges among the public health jurisdictions that will facilitate future outreach efforts to people living with HIV, and it provided a direct opportunity to clean up outdated HIV surveillance records, including updating vital status and current address.

As expected, this study saw that higher matching strength categories (eg, exact, very high, and medium high) observed higher rates of accuracy (>90%) with matches across jurisdictions, while lower matching strength categories (eg, low and very low) saw lower rates (15-30%) of accuracy with matches across jurisdictions (see Tables 3-5). It is worthy to note that lower matching categories still provided important data on potential matches for jurisdictions. Additionally, after manual review of the lower strength matches, this study found that lower strength matches remain valid for finding person matches. Higher-level matches may therefore be more readily accepted, while lower-level matches should still require manual verification. These higher matching categories are good for keeping updated surveillance records or for doing matching across different public health jurisdictions. The higher level matching strength categories (ie, exact, very high, and high) had very high levels of match validation with almost no false matches. This would support accepting an automated matching process that could be used either for more timely surveillance activities, allowing jurisdictions to identify individuals for interventions or to produce reliable statistical analyses of care patterns across jurisdictions without direct sharing of confidential identifying data and protected health information.

Additionally, lower matching categories are useful for improving matches. The lower matching strength categories (ie, low, very low) had low levels of match validation (15-30%), which would not be appropriate for routine matching and specifically not appropriate for automated real-time matching. However, they did produce initially large numbers of valid matches and could be utilized by jurisdictions to improve the completeness of their datasets, which would then improve later automated matches.

The importance of this effort for improving the effectiveness of surveillance data can already be seen in recent pilot efforts by jurisdictions to utilize surveillance data for public health action. For example, in VA, a pilot study of intervening with persons considered lost to care (n=43) found that 39% of these persons were actually in care, 21% were living out of state, and 7% were deceased. It appears that the majority of the in-care cases were categorized as lost to care due to incomplete lab reporting, although ongoing investigation continues of each case. These results translated into two-thirds of persons not requiring follow-up by VA D2C personnel, but because this information was not known to the surveillance team, time and effort was expended to locate and re-engage these persons. The matching method applied here can dramatically improve the timeliness and efficiency of public health action in the DC metropolitan region.

### Comparison to Traditional Surveillance Methods and Timeliness of Public Health Action

RIDR is an activity that relies on CDC to run its algorithm to identify potential matches across jurisdictions using the Soundex and other variables. Semi-annually, the CDC produces a list of potential matches for each jurisdiction to review with the other identified jurisdictions to ascertain who diagnosed the case. Then each jurisdiction exchanges information over the phone, updates the eHARS records, and gives the record a designation of either “same as” or “different than.” In contrast, our method identified persons already included in prior RIDR lists and also added to such lists new matches that were not previously included at a much faster rate than RIDR. Additionally, this method allowed for inclusion of recently updated data, unlike RIDR, which may include outdated information on vital status and residence.

### Addressing Barriers to Public Health Data Sharing

This novel data-sharing method also provided means for improved surveillance data and public health action, while it simultaneously addressed the six major barriers to public health data sharing as previously outlined by van Panhuis et al [3]:

1. *Technical:* According to van Panhuis et al, incompatible electronic record systems in multiple languages tend to prevent sharing public health data. This project employed the system that all public health jurisdictions use to report HIV information to CDC known as eHARS. The mode of connecting each local eHARS to the privacy device fell within the technical competence of each jurisdiction in consultation with the Georgetown University Information Services staff.
2. *Motivational:* The literature review found that different missions and local orientation undermined motivation for sharing information among public health jurisdictions. From the perspective of the public health jurisdictions in this project, the matching algorithm enabled finding persons otherwise lost to care, enhanced the value of available data, and established jurisdictions as pioneers—all strong motivating factors in their participation.
3. *Economic:* The literature review identified cost of partnering as a barrier to data sharing. Although staff from the jurisdictions invested many more hours in this project than the grant covered, they realized that, if successful, the matching algorithm could reduce time and labor to find persons in other jurisdictions from months to minutes, impractical to feasible, and unaffordable to affordable.
4. *Political:* According to van Panhuis et al, barriers of mistrust often prevent data sharing among public health agencies. Participating in this project encouraged the jurisdictions to exchange formal data use agreements. Beyond these formal agreements, however, the project work built a solid collaboration through a continuous series of project meetings, milestones, and major achievements over 2 years, including conferences hosted by Georgetown University in Washington, DC, on regional sharing of infectious disease data (January 2013) and the privacy device (November 2014), and presentations at the National Institutes of Health (NIH) “Harnessing Big Data to Halt HIV” conference (July 2015) and the National HIV Prevention Conference in Atlanta, Georgia (December 2015).
5. *Legal:* The literature review notes that, in many cases, incompatible laws, rules, and policies block data sharing. The jurisdictions in this project always had the legal right to share data for cases that involved other jurisdictions through separate data-sharing agreements. Nonetheless, the precise design of the technology enabled the algorithm to compare cases across jurisdictions without exposing data that should not be shared (ie, cases that did not involve other jurisdictions) either to other jurisdictions or to the third party facilitating the matching (Georgetown University).
6. *Ethical:* From a purely ethical perspective, we see our responsibilities with respect to this novel architecture and data flow in terms of data stewardship—the total process of investigating and safeguarding the ethical and privacy implications of recombining, reusing, repurposing, and reanalyzing multiple types of data from multiple sources with the explicit purpose of identifying and providing care to PLWH, especially those who, for various reasons, have dropped out of care [10]. However, the authors of the literature review focus on fairness of organizational work distribution in partnering for data sharing. Partnerships in data sharing often fail because some partners do more work than others and feel abused. From the perspective of the literature review, therefore, this project functioned as a true collaboration where the public health jurisdictions served as co-investigators, co-designers, data providers, and project beneficiaries. The actual data exchange fell within their purview and authority as public health jurisdictions with the right and responsibility to manage information about persons with HIV in their jurisdictions. Georgetown University did not view protected health information within the course of this study.

### Limitations and Future Directions of Study

While this method of matching people across eHARS databases in different jurisdictions saved time in comparison with more traditional methods, the manual validation process of this study was relatively time consuming. Therefore, future efforts should consider how to more effectively streamline this manual validation process. Also, although it was clear to the authors that the privacy device computed person matches across eHARS databases in a relatively short amount of time, while outside the scope of this initial study, future studies could perform a comprehensive assessment of the economic impact of implementing this technology in comparison with more traditional methods. Additionally, although the privacy platform provided high privacy assurance, one should note that with fewer resource constraints, more computational and mathematical power could be added to develop an even higher level of privacy assurance. Furthermore, no records were matched as medium low or low. This might indicate that parameters that defined such matching categories were insufficient for these purposes and should be revised for future projects. Moreover, it is important to underline that since this study considered all valid eHARS case records, its outcome is reflective of both historical and current patient migration in this metropolitan region from 1981 to 2015. This may or may not be aligned with current patient migration rates; therefore, future efforts should further examine the levels of migration over the last 3-5 years in the cross-jurisdictional DC metropolitan region. Although this study used RIDR information as part of the validation processes to check if the privacy device matches were indeed reported in pre-existing RIDR lists, future study directions could include a comprehensive evaluation to check if the opposite is also true—that all known RIDR matches can be detected using the privacy device. Similarly, while beyond the scope of this methods-focused study, future research efforts could also assess the overall impact of this data-sharing technology on public health reporting to CDC.

The person matching in this study was performed on all valid cases, ignoring any previously known information on migration and data sharing between jurisdictions. Future iterations should incorporate existing knowledge on interjurisdictional cases and also explore characteristics of individuals who migrate across state borders for HIV care to find patterns that can be helpful in identifying intervention points along the HIV care continuum. Exploring longitudinal cohort data from this geographic region may help supplement essential data on why people experience patient in- and out-migration in this region. In this regard, it may be useful to examine data from observational cohorts like the District of Columbia Metropolitan Women’s Interagency HIV Study—an ongoing prospective cohort study of HIV infection in women across Washington DC, Montgomery County, Maryland, and Northern Virginia [11]. It is also worthy to note that this study considered those who were already confirmed as HIV infected (as indicated by eHARS case record) and was not designed to address individuals who are unaware of their HIV status (ie, no eHARS record) or address those who were not matched.

This method can be used to help public health officials and their partners develop HIV care continuum models that better contextualize HIV in the United States for resource allocation purposes [12,13]. It can, for example, be used in future efforts comparing more traditional surveillance methods (eg, eHARS) with newer social media techniques (eg, Twitter, Google Flu Trends, HealthTweets) or in exploring patient in- and out-migration, which remains a large knowledge gap in HIV epidemiology [14-16]. Moreover, since large metropolitan regions experience high levels of person movements, such areas may provide fruitful grounds for further examination of mobility in HIV care using this novel approach [17]. Lastly, this technology is applicable to public health data sharing outside of HIV disease surveillance, and the authors have already began to explore applications to other infectious disease data.

### Conclusion

Using a novel technology and interdisciplinary and public-private partnership, this study effectively addressed how to improve HIV surveillance data for public health action. This approach can provide a more effective bridge between data and care in public health and may be applied to other purposes.

## Appendix

Multimedia Appendix 1 Algorithm development.

## Abbreviations

AIDS: acquired immune deficiency syndrome
CDC: Centers for Disease Control and Prevention
D2C: data to care
DC: District of Columbia
DC-CFAR: District of Columbia Center for AIDS Research
DOB: date of birth
eHARS: Enhanced HIV/AIDS Reporting System
HIPAA: Health Insurance Portability and Accountability Act
HIV: human immunodeficiency virus
MD: State of Maryland
PLWH: people living with HIV
RIDR: Routine Interstate Duplicate Review
SSN: social security number
STATENO: state number
VA: Commonwealth of Virginia
WIHS: Women’s Interagency HIV Study
