# Supplementary material for: Improving HIV Surveillance Data for Public Health Action in Washington, DC: A Novel Multiorganizational Data-Sharing Method
Source: JMIR Public Health Surveill. 2016 Jan 15;2(1):e3. doi: 10.2196/publichealth.5317 (PMC4869245; doi:10.2196/publichealth.5317)
Supplement: Multimedia Appendix 1 [file publichealth_v2i1e3_app1.pdf]

Multimedia Appendix 1

I. Detailed algorithm development, and hardware and system configuration methods

eHARS cases definitions

For this study, cases included were explicitly defined as: all persons with a record in the eHARS person-view table with status_flag = A (active record) or W (warning), and dx_status = 1 (adult HIV), 2 (adult AIDS), 4 (pediatric HIV), or 5 (pediatric AIDS), and the state identification number or STATENO (jurisdiction-specific identification variable) not missing.

Algorithm development

The Ada programming language was selected for algorithm implementation. The main subprogram of the algorithm was as follows. More details on the computational aspect of our study methods may be found in the reference section.[17]

```
procedure Main is
begin
Initialize; -- Erase/build directories & logs
            loop
            if Update then      -- Check for new data files
                 Analyze;    -- Search for matches
                 Report;     -- Report matches
                 Clear;      -- Clear matches
            end if;
            delay scan_time;
         end loop;
end Main
```
